# Unveiling the relationship between pain and bacterial load in venous ulcers with implications in targeted treatment

**DOI:** 10.1016/j.jvsv.2025.102213

**Published:** 2025-02-19

**Authors:** Alisha R. Oropallo, Priscilla J. Lee, Amit Rao, Micaela D. Gray

**Affiliations:** a Department of Vascular and Endovascular Surgery, Comprehensive Wound Healing and Hyperbarics, Lake Success, NY; b Donald & Barbara Zucker School of Medicine at Hofstra/Northwell, Hempstead, NY; c MolecuLight, Inc., Toronto, Ontario, Canada

**Keywords:** Bacterial load, Fluorescence imaging, Pain, Quality of life, Venous leg ulcers

## Abstract

**Objective:**

The relationship between bacteria and venous ulceration pain is well-established and primarily attributable to inflammatory pathways. Fluorescence imaging detects clinically significant bacterial loads and biofilm in real time at the bedside, informing its elimination in an objective manner. We sought to explore the regional co-localization of bacterial fluorescence signals and patient-reported venous ulceration pain, and if objectively targeted bacterial removal can reduce wound-associated pain.

**Methods:**

We evaluated 46 adults with venous ulceration of the lower extremity self-reporting a wound-associated pain score of ≥4 on a scale of 1 to 10. Before any treatments were performed (eg, debridement), patients rated their pain during the study visit, and fluorescence images were captured. Regions of pain and positive fluorescence signals were sketched onto a printed wound image. Fluorescence imaging was repeated post procedurally, and patients rerated their pain either at the end of the study visit or over the phone the following day. Semiquantitative analysis involved visual estimation of the percentage overlap between regions of fluorescence and pain in the wound bed. Wilcoxon matched pairs signed rank tests and Mann-Whitney *t* tests assessed changes in pain scores post procedurally.

**Results:**

Fluorescence from elevated bacterial loads and biofilm was present in every venous ulcer assessed, usually covering ≤50% of the wound bed and commonly colonizing the wound edges. Regions of pain were more extensive than regions of fluorescence within the wound bed, and some degree of overlap was identified in 40 of 46 patients (87%). This overlap was often substantial (29 patients with >25% overlap and 16 with >50% overlap). Overall mean pain scores were 8.17 before the procedure and 6.87 after the procedure, corresponding with a 1.30-point reduction that was highly statistically significant (*P* < .0001). Pain score reduction was higher when patients rerated their pain 1 day after debridement (3.40-point reduction; *P* = .004).

**Conclusions:**

We observed that fluorescence signals from clinically significant bacterial colonization and biofilms were commonly present in painful venous lower extremity ulcerations. Regions of patient-reported pain and positive fluorescence frequently overlapped, suggesting a relationship between the two. Wound-associated pain scores were significantly and immediately reduced after objectively targeted bacterial removal via real-time fluorescence imaging, with an even greater reduction observed by the next day. Understanding the association between chronic bacterial presence and pain in venous ulcers can inform treatment and management strategies, potentially enhancing patient quality of life and satisfaction, promoting healing, and reducing complications.

**Article Highlights**

• **Type of Research:** Single-center prospective observational study
• **Key Findings:** Targeted bacterial removal using real-time fluorescence imaging in 46 patients reduced patient-reported venous ulceration pain scores by 1.30 points (*P* < .0001). Regions of pain were frequently colocalized with bacterial fluorescence, with 87% of patients showing some degree of overlap.
• **Take Home Message:** There seems to be a regional colocalization between venous ulceration pain and bacterial loads identified by fluorescence imaging, where targeted bacterial removal effectively reduced patient-reported pain.

Venous ulcers are severe, late-stage clinical manifestations of chronic venous insufficiency, generally described as open skin lesions of the lower extremities that occur in areas with venous hypertension and/or reflux.^1^^,^^2^ In the United States, chronic venous ulcers are present in approximately 1% to 3% of people aged 18 to 64 years, and this rate increases to 4% for adults >65 years.^3^^,^^4^ It has been estimated that roughly 10% of people will develop a chronic venous wound in the course of their lifetime.^5^ There is a growing concern for the prevalence of venous leg ulcers driven by surging rates for risk factors such as elevated body mass index, venous thromboembolism, venous disease, diabetes, and sedentary lifestyles.^6^, ^7^, ^8^

Chronic venous ulcers are debilitating and painful, often having a severe impact on people's well-being and quality of life. Symptoms can include burning pain, itchiness, tingling, throbbing, cramps, and dull heaviness with >80% of patients reporting acute or chronic wound-associated pain.^9^, ^10^, ^11^, ^12^, ^13^ Venous ulceration pain is associated with sleep disturbance, disrupted daily functioning, and feelings of hopelessness, powerlessness, and despair.^9^ One study reported that the pain was significant enough that one-third of patients were unable to attend their wound care appointments.^14^ Venous ulceration pain is often worsened by local treatments and management; patients describe experiencing the most excruciating pain during dressing changes.^15^ Although pharmacological interventions are available for the management of wound-related pain, they are typically insufficient for total pain relief and may be associated with adverse side effects.^16^^,^^17^ There has been a sustained effort within the scientific community to determine the underlying causes of venous ulceration pain. One proposed model suggests that inflammation may play a role in the onset of chronic wound pain.^9^ Therefore, it is important to consider the link between wound biofilms, their contribution to a proinflammatory state and its associated pain.

Bacterial biofilms are commonly found in venous ulcers and are associated with a heightened inflammatory response and prolonged healing times.^11^^,^^18^^,^^19^ Biofilms have an extracellular matrix that triggers the activation of macrophages and neutrophils. These immune cells release proinflammatory cytokines to keep the wound in a self-perpetuating state of inflammation and to protect the bacteria from the host's immune response and antibiotics.^11^^,^^19^, ^20^, ^21^ It is thought that inflammatory exudate serves as a nutrient source for bacteria. Proinflammatory cytokines are also known to stimulate nociceptors, which are responsible for the detection and transmission of pain.^22^^,^^23^ Thus, pain is well-established as a clinical manifestation of inflammation. Although studies have explored the associations between bacterial colonization and inflammatory pain in chronic conditions, such as within the gut microbiome, few have assessed the impact of bacteria on venous ulceration pain.^24^^,^^25^

Fluorescence imaging devices (MolecuLight Inc., Toronto, Ontario, Canada) offer a noninvasive, point-of-care approach to detect and visualize bacteria and biofilm in wounds. Red fluorescence signals mark area with bacterial burdens exceeding 10^4^ CFU/g in skin wounds.^26^, ^27^, ^28^ Owing to its ability to provide immediate feedback, the MolecuLight *i:X* and DX fluorescence imaging devices facilitate real-time monitoring and objectively guided bacterial infection management.^29^

This novel study aimed to evaluate the association between high bacterial colonization and wound-associated pain in venous ulceration. Using Moleculight *i:X* for bacterial detection, we hypothesize that pain would coincide with bacteria and biofilm presence and that targeted debridement would result in pain reduction. Understanding the association between bacteria and pain in venous ulcers can inform treatment and management strategies, thus impacting both clinical and patient outcomes.

## Methods

### Study design

Patients were recruited from Northwell Health Comprehensive Wound Healing and Hyperbarics Center, New York. In this prospective single-center cohort study, consecutive patients with active venous ulceration of the lower extremity were screened and recruited into the study if they self-reported at the study visit a wound-related pain score of ≥4 (Numerical Pain Score Assessment, 1-10). Up to one ulcer per leg, per patient, was eligible for inclusion. Arterial insufficiency was ruled out by palpating pulses on the lower extremities. Patients reporting pain scores of <4 or who were unable or unwilling to report pain scores were excluded during initial screening. Neuropathic patients (confirmed via Semmes-Weinstein test) were also excluded to reduce confounding factors and focus the investigation on nociceptive pain arising from inflammation. This clinical investigation was performed in accordance with the principles of Good Clinical Practice and the Declaration of Helsinki. Institutional review board approval was obtained from Northwell Health institutional review board. Each subject's consent was obtained before participation in any study conduct, and all participants agreed to their case details and images being published.

### Data collection

Standard and fluorescence wound images were captured using the MolecuLight *i:X* device. The device accurately detects bacterial loads of >10^4^ CFU/g, with a positive predictive value of 95%.^30^ All fluorescence images were captured by the research coordinator, who had received training from the device manufacturer on proper image acquisition techniques and had several years of experience in capturing and interpreting fluorescence images in a clinical setting. Furthermore, the examination room lights were turned off during fluorescence image capture to ensure image accuracy. Data regarding patient-reported pain, bacterial infection management, and the research coordinator's interpretation of the fluorescence images were collected using a case report form (CRF). Patient demographics and wound characteristics were obtained from the Northwell Health Electronic Medical Record Allscripts Touchworks and Net Health Tissue Analytics.

### Study protocol

During each study visit, the wound was wiped with saline and gauze to remove any superficial debris and then a standard image was captured. The patient was then asked to point to area(s) of pain, rate that pain on a scale of 1 to 10 (no pain to worst pain imaginable), and then sketch the area(s) of pain directly on a printed, standard image of their wound. In cases where the patient was unable or unwilling to mark up the image, the study coordinator drew the area(s) of pain using direction from the patient. Using wound software system, Tissue Analytics, wound measurements including square surface area were calculated per standard protocol. Next, the research coordinator captured and interpreted fluorescence images of the wound at the bedside and sketched the areas of red and/or cyan fluorescence on the printed image. Wound care then proceeded as per usual, where the provider could use any available treatment deemed medically necessary. Providers were not blinded to the fluorescence imaging findings and could use this information to inform their care plan or treatment(s). The research coordinator recorded if and how the information from fluorescence imaging impacted the provider's clinical decisions and whether the fluorescence was completely removed.

Finally, fluorescence images were captured again after all treatment(s) for that visit were performed and before the wound was re-dressed. For the first 37 venous ulcers screened in, the patient was asked at the end of the study visit to re-rate their pain on a scale of 1 to 10 (no pain to worst pain imaginable), generating a postprocedural pain score. For the final 10 venous ulcers assessed, patients provided their postprocedural pain scores to the research coordinator over the phone the day after the study visit. This protocol adjustment was made to provide further insight into the possibility of delayed pain relief, because certain procedures such as debridement are inherently painful. No pain control was administered during treatment, allowing for the assessment of untreated pain levels after the procedure.

### Data processing and analysis

The CRFs, standard and fluorescence images, and annotated wound schematics were uploaded to a secure cloud-based portal for review and storage. Data from the CRFs were transcribed into an Excel spreadsheet and scrubbed of personal health information to protect patient confidentiality. The wound schematics were annotated as follows: red to indicate area(s) of red fluorescence, green to indicate area(s) of cyan fluorescence, and black to indicate area(s) of patient-reported pain.

**Fig 1:**
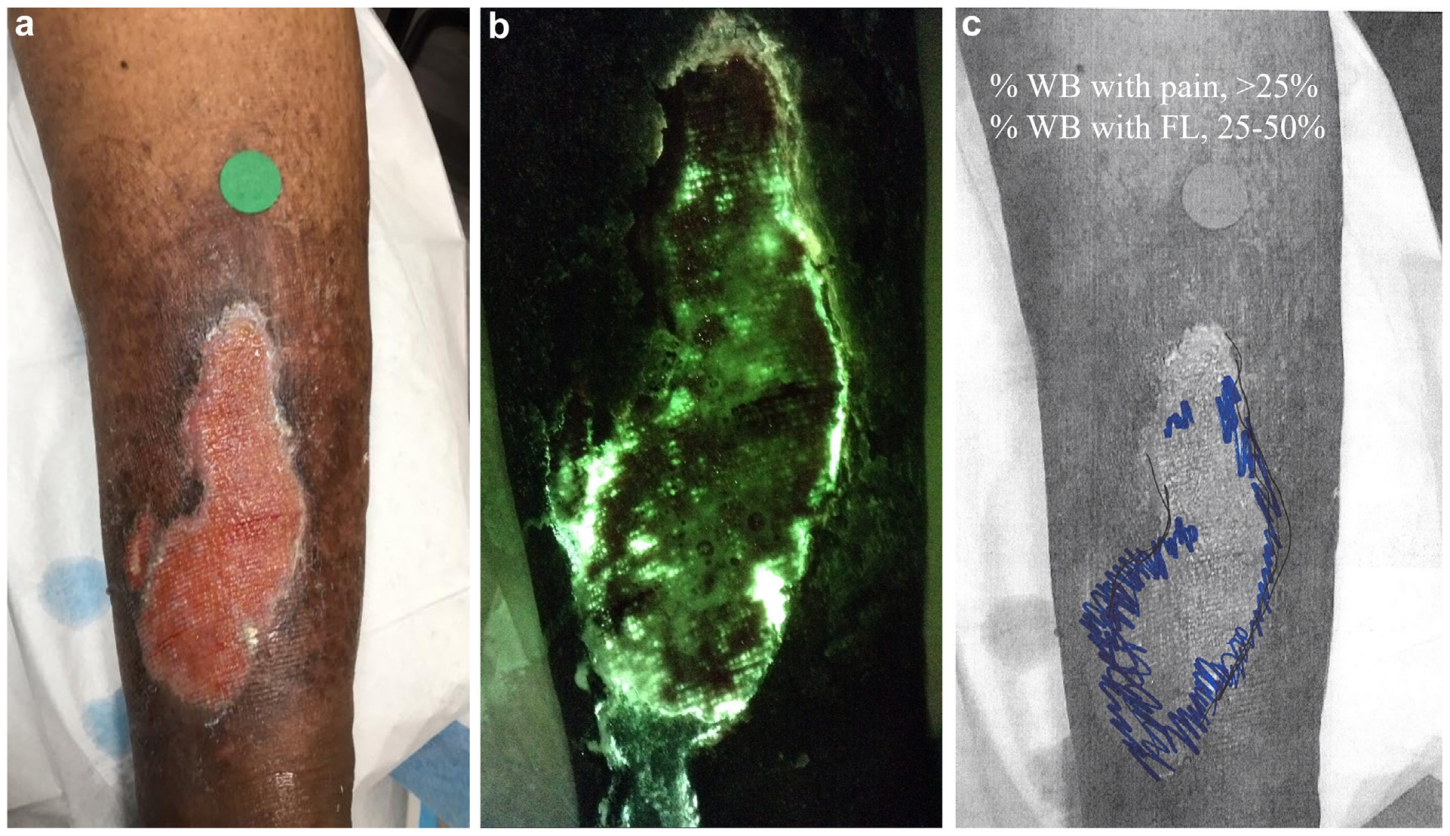
**(A)** Standard wound image. **(B)** Fluorescence image showing *Pseudomonas aeruginosa* at loads of >10^4^ colony-forming units per gram. **(C)** Wound schematic annotated with regions of pain (*black*) and bacterial fluorescence (*blue*). Per the schematic, the percentage of the wound bed with pain or bacterial fluorescence was <25% and 25%-50%, respectively.

Using the annotated images, the percentage of wound bed with patient reported pain and the percentage of wound bed with red and/or cyan fluorescence were visually estimated (Fig 1). The following thresholds for this estimation were used: 0%, ≤25%, ≤50%, ≤75%, and ≤100%. Regions of fluorescence and patient-reported pain were correlated semiquantitively by estimating the percentage overlap also using these thresholds. It was noted if there was pain and/or fluorescence in the periwound region, although fluorescence signals outside the wound bed were not included in the visual percentage estimations. Both the standard and fluorescence images were consulted during the visual estimations as needed to clarify image annotations.

### Statistics

All statistical analyses were performed using GraphPad Prism v10.2.3. Two-tailed Wilcoxon matched-pairs signed rank tests assessed the statistical significance of changes between preprocedural and postprocedural pain scores. Mann Whitney *t* tests compared the preprocedural and postprocedural pain scores between the cohorts report their scores at the end of the study visit or over the phone the next day.

## Results

### Cohort characteristics

Of the 67 patients screened, a total of 46 met the study inclusion criteria. The most common cause of exclusion was pain scores of <4. Patient- and wound-level cohort characteristics are reported in Table I. Patients were primarily Black or White with ages ranging from 41 to 94 years. Their ulcers were all venous origin diagnosed by positive venous reflux studies and were primarily located on the malleolus. Circumferential wounds were included, and ulcer size ranged from 0.7 to 74.1 cm^2^, 19.7 cm^2^ on average. Approximately one-quarter of the patient cohort (12/46) reported the previous use of analgesics at the time of study inclusion.

**Table I:** Patient- and wound-level cohort characteristics

Patient level	No. (%)	Wound level	No. (%)
Sex		Ulcer etiology	
Male	25 (54.3)	Venous	46 (100)
Female	21 (45.7)	Other	0 (0)
Age		Surface area (cm^2^)	
Average ± SD	70 ± 13	Average ± SD	19.7 ± 18.5
Median (quartiles)	73 (63-78)	Median (quartiles)	13.6 (3.7-32.4)
Race		Analgesic medication^a^	
Asian	3 (6.5)	Yes	12 (26.1)
Black	22 (48.9)	No	34 (73.9)
White	17 (37.0)		
Other/mixed race	4 (8.7)		
Neuropathic			
Yes	0 (0)		
No	46 (100)		

*SD,* Standard deviation.

a Patient-reported taking analgesics for index wound-related pain management before the study visit.

### Patient-reported pain

Regions of pain were reported throughout the wound bed, wound edge, and/or in the periwound. We found that 32.6% of patients (15/46) reported pain in the periwound region, and pain was commonly noted along the wound edges (36/46 patients [78.3%]).

Patient-reported pain scores are presented in Table II. Preprocedural pain scores were reported by all patients before any treatments were performed. Postprocedural pain scores were reported by patients either immediately after wound treatments at the end of the study visit or the following day during a follow-up phone call with the research coordinator. Overall, pain scores decreased for 24 patients (52.2%), increased for 4 patients (8.7%), and stayed the same for 18 patients (39.1%).

**Table II:** Patient-reported pain scores relating to lower extremity ulceration of venous etiology

Cohort	Preprocedure “before” pain score	Postprocedure “after” pain score	Change in pain score	*t* Test
All patients (n = 46)				
Mean ± SD (95% CI)	8.17 ± 1.76 (7.65 to 8.70)	6.87 ± 2.57 (6.11 to 7.63)	−1.30 (−0.70 to −1.91)	*P* < .0001
Postprocedural pain score reported immediately during the study visit (n = 36)				
Mean ± SD (95% CI)	8.22 ± 1.74 (7.63 to 8.81)	7.50 ± 2.06 (6.65 to 9.35)	−0.72 (−0.17 to −1.27)	*P* = .012
Postprocedural pain score reported over phone the following day (n = 10)				
Mean ± SD (95% CI)	8.00 ± 1.89 (6.80 to 8.20)	4.60 ± 3.03 (2.43 to 7.76)	−3.40 (−1.96 to −4.84)	*P* = .004

*CI,* Confidence interval; *SD,* standard deviation.

For all 46 patients, the overall mean pain scores were 8.17 before the procedure and 6.87 after the procedure, corresponding with a 1.30-point reduction in patient-reported pain that was highly statistically significant (*P* < .0001). A greater point reduction was observed among the group that reported their postprocedural pain scores over the phone the following day (n = 10). The mean pain scores in this group decreased from 8.00 to 4.60, corresponding with a statistically significant 3.40-point pain score reduction (*P* = .004).

### Bedside real-time fluorescence imaging

**Fig 2:**
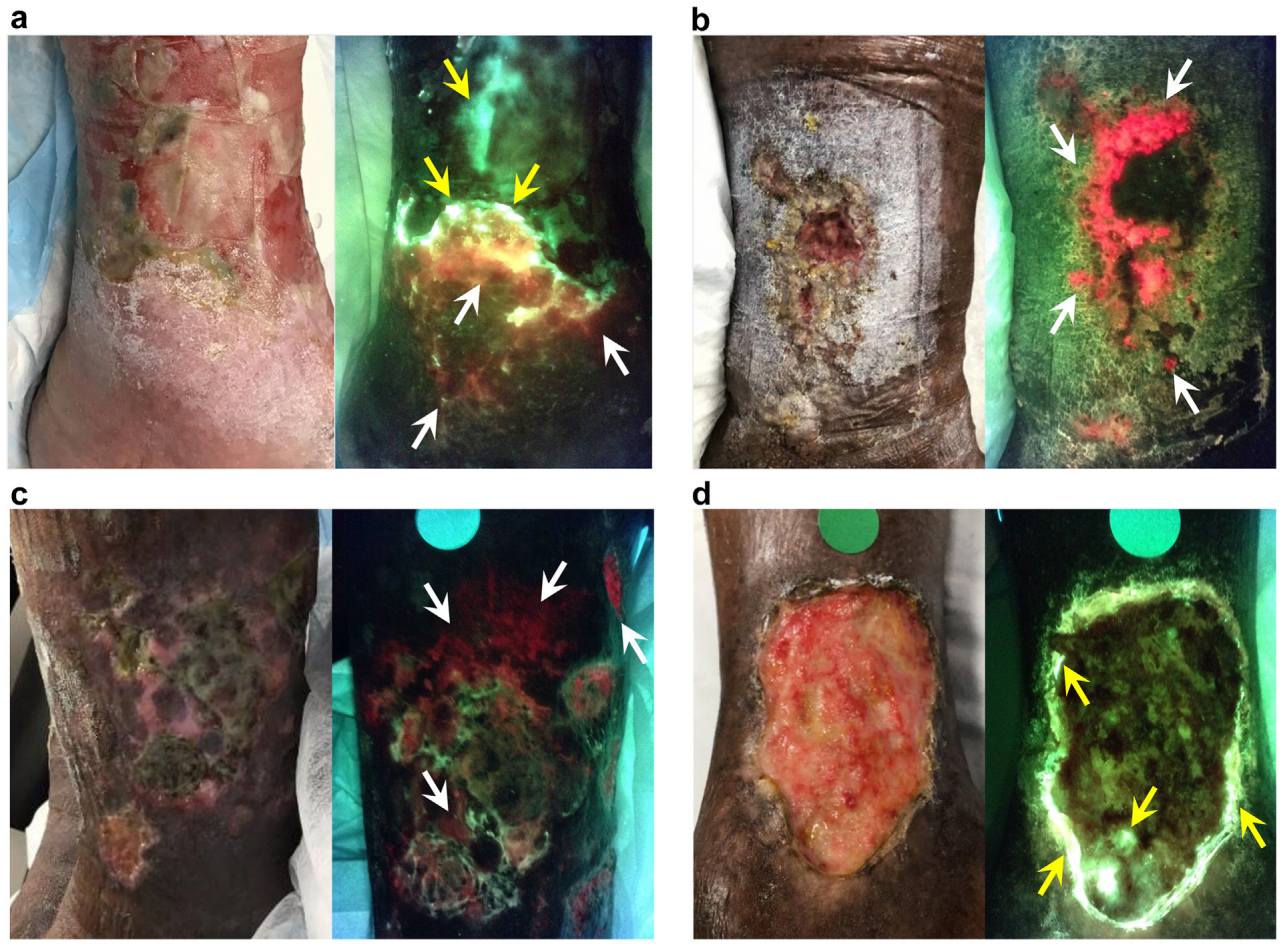
**(A-D)** Standard (left) and fluorescence (right) image pairs. *White* and *yellow arrows* point to regions of *red* and *cyan* fluorescence, respectively, indicative of bacterial colonization (>10^4^ CFU/g). *Red* fluorescence (also *yellow*, *blush*, and *orange*) signals the presence of most bacterial species commonly found in chronic wounds, and cyan fluorescence signals the presence of *Pseudomonas aeruginosa*, specifically.

Significant bacterial colonization/biofilms were present in every venous ulcer assessedwith fluorescence signals apparent either in the wound bed, wound edge, and/or periwound region. All but one exhibited positive fluorescence along the wound edge (45/46 [97.8%]). Twelve ulcers exhibited red fluorescence only (25.5%), 17 exhibited cyan only (36.2%), and 18 exhibited both red and cyan fluorescence (38.3%). Example fluorescence images are provided in Fig 2.

Bacterial colonization/biofilms unveiled by fluorescence imaging commonly impacted wound treatment. Fluorescence imaging led the provider to select a wound cleansing solution using a scrubbing technique^31^ (5/46 wounds), to cleanse over a larger area than planned (21/46 wounds), to debride over a larger region than planned (12/46 wounds), or to target debridement to regions of fluorescence (16/46 wounds). Providers used sharp excisional debridement techniques in addition to mechanical wound cleansing.

Per standard practice at Northwell, providers re-imaged after cleansing/debridement to determine its efficacy. Postprocedural imaging confirmed that fluorescence signals were eliminated completely from 19 of 46 ulcers (41.3%). In two cases where the bacteria could not be removed completely during the office visit, antibiotics were prescribed because these patients had been recently discharged from the hospital for cellulitis infection. When comparing the reduction in mean pain score between patients where the fluorescence could or could not be eliminated (−1.1 and −1.5 points, respectively), there was no significant difference (*P* = .4362). There was also no difference in pain score reduction in relation to the patient's use of analgesics (−1.0 and −1.4 points, respectively; *P* = .3870).

### Colocalization of pain and fluorescence imaging

We performed a semiquantitative analysis to investigate the colocalization of patient-reported pain and fluorescence signals from bacteria/biofilm. As described in detail in the study methods, regions of pain and fluorescence were each categorized into discrete thresholds depending on their relative area as compared with the wound bed (0%, ≤25%, ≤50%, ≤75%, or ≤100%). For example, if the patient noted pain over the entire wound bed but there were only a few specks of fluorescence at the wound edge, the pain would be categorized as ≤100% and the fluorescence as ≤25%.

**Fig 3:**
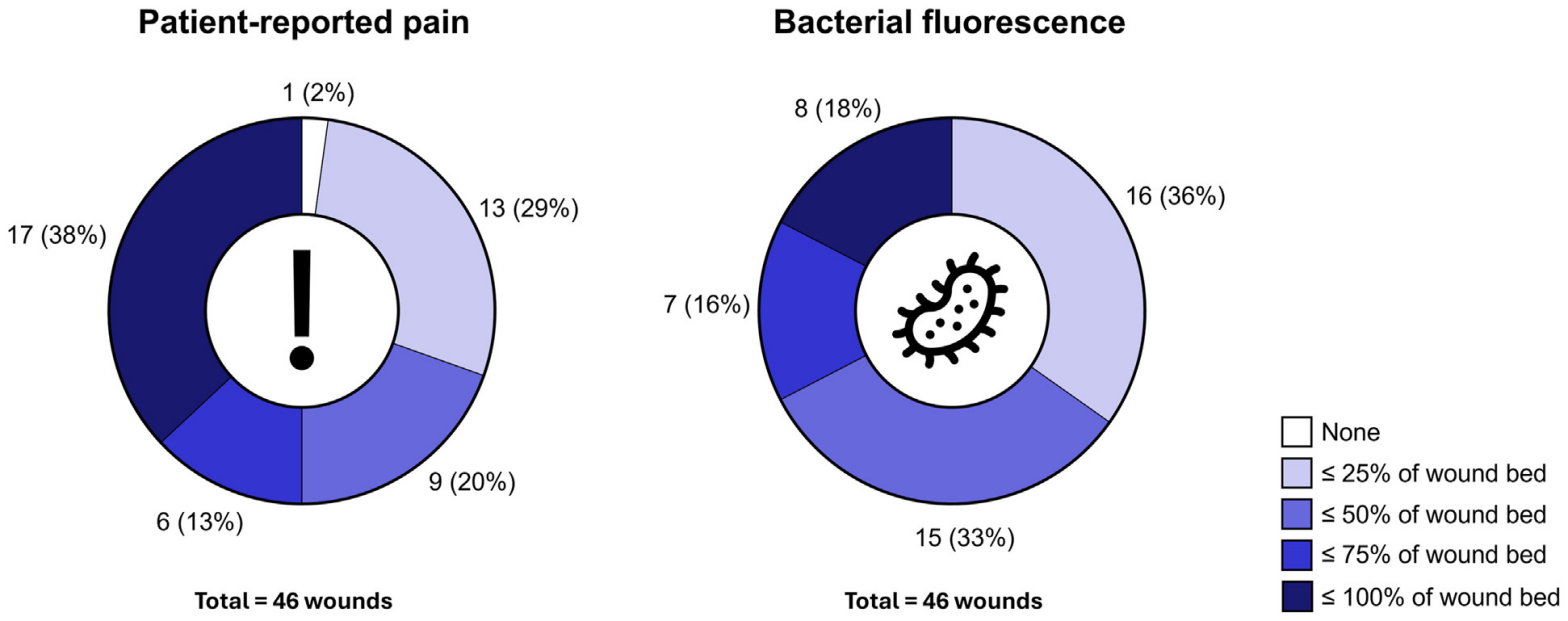
Estimated relative abundance of regions of patient-reported pain and fluorescence from bacteria/biofilm among venous leg ulcers.

Regions of patient-reported pain were generally larger than regions of bacterial fluorescence (Fig 3). One-half of the patients (23/46) reported pain that covered ≥50% of the wound bed, and 17 of 46 (36.9%) reported pain that covered anywhere from 75% to 100%. One patient reported isolated periwound pain, classified as 0% of the wound bed. Most patients exhibited fluorescence signals covering ≤50% (31/46 [67.4%]) of the wound bed. Fifteen of 46 patients (32.6%) displayed fluorescent signals covering >50% of the wound bed, and 8 of 46 patients (17.4%) displayed fluorescence extending over 75% of the wound bed. There were no significant differences in pain and bacterial load between medial and lateral malleolar venous ulcers.

**Fig 4:**
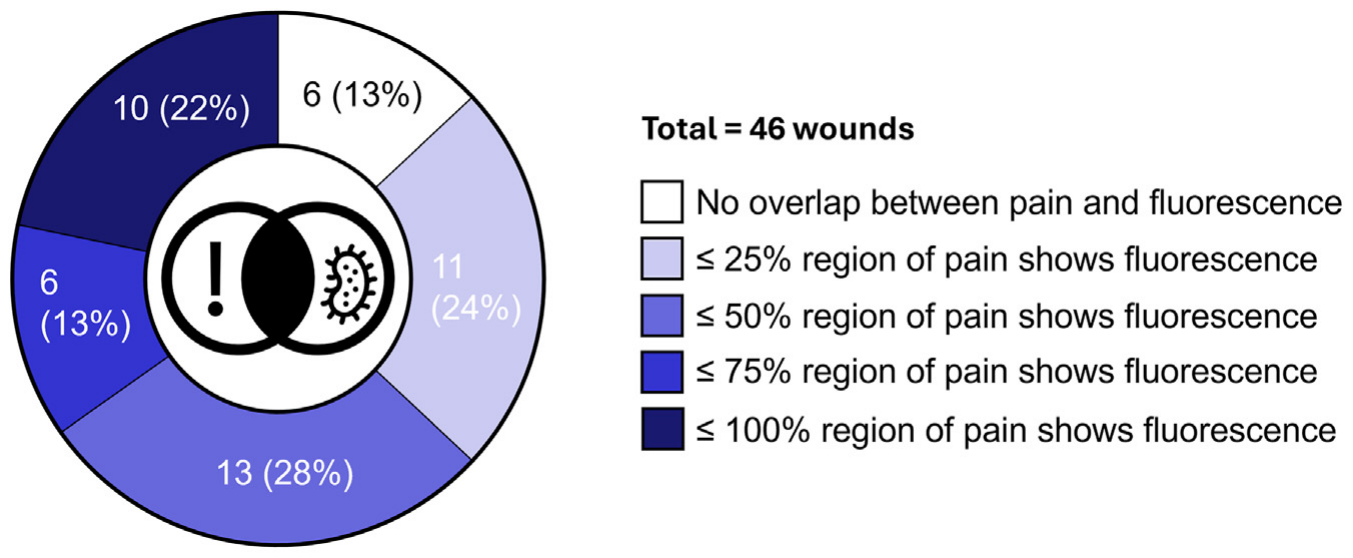
Estimated proportions of regions of patient-reported pain with fluorescence signals.

Some level of overlap between regions of patient-reported pain and fluorescence from bacterial colonization/biofilms was noted in 40 of the 46 venous ulcers studied (87%). We measured the extent of this overlap by visually estimating the proportion of painful regions that contained fluorescence signals (Fig 4). When considering only cases where overlap was observed, the fluorescence signals usually covered ≥25% of the regions corresponding with patient-reported pain (29/40 cases [72.5%]). For 16 patients (40.0%), more than one-half of the area of patient-reported pain showed fluorescence, and for 10 patients (25.0%) that overlap was estimated to be 75% to 100%. Thus, there was usually substantial overlap between patient-reported pain and fluorescence regions. However, no overlap was noted for 6 of the 46 patients studied (13.0%).

## Discussion

Chronic, hard-to-heal venous ulcers present significant challenges in health care systems owing to their prolonged healing times, which increases the risk of infection and adverse effects on psychosocial well-being.^1^^,^^3^ Preclinical research has provided insights into the relationship between bacteria and pain in these wounds, primarily attributable to inflammatory pathways and biofilm formation.^19^, ^20^, ^21^^,^^32^ Biofilms are aggregates of micro-organisms that embed themselves in an extracellular polymeric matrix, creating a physical and functional barrier to guard against the host's immune system responses.^19^^,^^21^ They also stimulate proinflammatory cytokines to maintain a self-perpetuating inflammatory state, contributing to wound chronicity. Cytokines, such as interleukin (IL)-1, IL-6, and tumor necrosis factor-ɑ, stimulate nociceptor receptors and contribute to the overall perception of pain.^22^^,^^23^ Bacteria that produce biofilms are also involved in the activation of matrix metalloproteinases and neutrophils, which release enzymes that damage surrounding tissues.^32^ Tissue degradation in chronic wounds intensifies discomfort and hinders wound healing. This persistent process ultimately results in extended periods of pain for patients, highlighting the need for effective interventions and management strategies.

Although considerable literature has reported on the association between bacteria and pain, few studies have investigated the role of bacterial elimination in pain management, even less so in venous ulcers. We observed a statistically significant reduction in patient-reported pain in association with their venous ulcers when using the MolecuLight *i:X* imaging device to target bacterial removal and to inform wound management (1.30-point reduction; *P* < .0001). Fluorescence images guided cleansing and debridement of venous ulcers, providing greater precision in removing debris while minimizing damage to healthy tissues. We propose that this targeted method of bacterial elimination reduced bacterial-associated inflammation, resulting in a subsequent decrease in reported wound pain. These results have further strengthened our confidence in using advanced imaging-informed techniques to guide debridement and cleansing with the added benefit of addressing venous ulcer pain.

Overall, the reduction in pain scores was more pronounced when assessed 1 day after treatment compared with measurements taken immediately afterward (3.40-point vs 0.72-point reduction, respectively). This finding may be explained by the confounding effect of the procedure-related pain, which had subsided 1 day later. This effect has been described in multiple studies on venous ulceration pain, which is frequently worsened by the abrasiveness of cleansing and dressing techniques.^15^^,^^33^^,^^34^ Psychosocial factors such as anxiety and fear may also contribute to a heightened pain experience during the wound care appointment.^9^ It can be reasonably assumed that pain scores reported the day after treatment provide a more accurate assessment of the effectiveness of targeted wound management in reducing pain, because these scores are less likely to be influenced by the physical discomfort or psychological factors associated with wound care treatment.

In addition to the observed pain reduction with fluorescence-informed wound management, our results also demonstrated a substantial overlap between regions of patient-reported pain and fluorescence, with 40 of the 46 venous ulcers (87%) exhibiting overlap. This result is consistent with the established understanding of the relationship between high bacterial loads/biofilms and pain in chronic wounds.^11^^,^^19^, ^20^, ^21^ Elevated bacterial loads provoke strong inflammatory responses, which contribute to higher pain levels. The high degree of colocalization further emphasizes the importance of addressing bacterial presence in venous ulcers for effective pain management. An intriguing aspect of our findings is the observation that regions of patient-reported pain were more extensive than regions of fluorescence. This finding is not entirely unexpected; wound-associated pain is understood to be influenced by a multitude of factors beyond pathogen-mediated inflammation.

However, pain is a subjective and complex manifestation of several, often concurrent, factors. In wound pain, tissue-related complications, infection, vascular insufficiencies, mechanical trauma, and environmental factors contribute to its manifestation.^9^^,^^10^^,^^13^ Woo et al^9^ also identified factors such as moisture balance, psychosocial well-being, and patient expectations as significant contributors to pain perception. It is important to recognize the subjective nature of pain and the variability in individual tolerance, which affects reported pain levels, even with standardized scales. Although imaging techniques can measure bacterial presence objectively, they contrast with the personalized experiences of pain that are influenced by cognitive, psychosocial, and contextual factors.

The results of this study further cement our belief that clinicians should consider implementing fluorescence imaging as a viable method in their clinical approach to managing chronic venous ulcers. There are limited technologies currently available that can provide point-of-care guidance for the visualization of bacteria in wounds. Only the MolecuLight *i:X* is validated to detect bacteria/biofilm reliably at clinically significant levels at the bedside. Traditional microbiological sampling is costly and time consuming, provides delayed results, and is unable to provide locational information on the extent of bacterial spread on a wound bed, which would aid wound cleansing and debridement significantly.^34^, ^35^, ^36^ In contrast, handheld fluorescence imaging devices permit a noninvasive, point-of-care approach to inform wound care in real time.^29^^,^^37^^,^^38^ Our results further demonstrate that imaging-informed cleansing and debridement are substantially effective at completely eliminating fluorescence signals (41.3% of venous ulcers). Moreover, a significant decrease in wound pain was observed regardless of whether bacteria were eliminated completely and there was no significant difference in pain score reduction between wounds with complete bacterial removal and those with residual bacteria. This finding suggests that complete bacterial removal is not required for patients to benefit from the precision and visual feedback provided by fluorescence wound imaging. Therefore, although the fluorescence imaging allows for visualization of areas with higher bacterial loads, its role in pain reduction may lie more in providing targeted visual feedback for more precise treatment rather than for eliminating bacteria completely.

There are several study limitations that must be acknowledged. First, the relatively small sample size may limit the generalization of our results. Moreover, data collection was limited to treatment during a single appointment, which does not account for the full scope of wound management. Longitudinal studies should be conducted to establish the role of imaging-informed treatment in the context of consistent, multidisciplinary management of chronic venous ulcers. We did not explore data on systemic markers of infection, specific bacterial strains, their level of invasiveness, or the type of venous reflux, which may have provided further insights. Including patients with pain scores of <4 and comparing wounds with lower and higher pain scores could have also strengthened the validity of our findings. These factors should be incorporated into future studies to provide more comprehensive insights into the relationship between pain and bacterial presence in venous ulcers. Despite these limitations, we believe that the Moleculight *i:X* imaging device offers a practical method to enhance the management of venous ulcers through its noninvasive, real-time fluorescent visualization.

## Conclusions

This prospective cohort study provides valuable insights into the relationship between bacterial presence and pain in lower extremity venous ulcers. We observed a substantial spatial overlap between areas of bacterial presence and patient-reported pain, and removal of fluorescence signals from bacteria/biofilms was associated with a significant reduction in pain scores. The pain experienced by patients with venous ulcers is a significant clinical concern and fluorescence imaging may offer a promising approach to addressing this issue. In our research, the application of fluorescence imaging proved beneficial in informing our cleansing and debridement techniques for pain management. Given the limited therapeutic options available for addressing ulceration-associated pain, clinicians should explore the use of fluorescence imaging to optimize wound care and improve patient outcomes.

## Author contributions

Conception and design: AO, AR, MG

Analysis and interpretation: AO, PL, MG

Data collection: AR

Writing the article: PL, AR, MG

Critical revision of the article: AO, PL, MG

Final approval of the article: AO, PL, AR, MG

Statistical analysis: MG

Obtained funding: Not applicable

Overall responsibility: AO

## Funding

None.

## Disclosures

M.D.G. is employed at MolecuLight, Inc., Toronto, Ontario, Canada.
